# The Stability of Nd Hydroxyl Complexes at Near Neutral to Alkaline pH from 25 to 75 °C: Implications for Monazite Solubility in Hydrothermal Aqueous Fluids

**DOI:** 10.1021/acsearthspacechem.5c00328

**Published:** 2026-07-07

**Authors:** Hannah J. Han, Alexander P. Gysi

**Affiliations:** † New Mexico Bureau of Geology and Mineral Resources, New Mexico Institute of Mining and Technology, 801 Leroy Place, Socorro, New Mexico 87801, United States; ‡ Department of Earth and Environmental Science, New Mexico Institute of Mining and Technology, 801 Leroy Place, Socorro, New Mexico 87801, United States

**Keywords:** rare earth elements, hydrolysis, UV−vis spectrophotometry, pH, REE transport, monazite

## Abstract

Speciation of rare earth elements (REE) in aqueous fluids controls their mobilization during fluid-rock interaction. Thermodynamic modeling provides important insights into the factors controlling REE mobility and fractionation in aqueous fluids, in which aqueous REE hydroxyl complexes are particularly important across a broad pH range. However, the stability of these REE hydroxyl complexes remains poorly determined due to limited experimental data above ambient temperature. In this work, UV–vis spectrophotometry experiments were conducted to determine the cumulative hydrolysis constants for neodymium (Nd) from 25 to 75 °C in near-neutral to alkaline solutions. The color indicator *m*-cresol purple was used to determine *in situ* pH. Alkaline NaOH-bearing aqueous solutions were doped with varying initial Nd concentrations (0 to ∼0.155 mmol/kg), which resulted in the release of protons (H^+^) and pH decrease during hydrolysis of Nd^3+^ according to Nd^3+^ + *n*H_2_O = Nd­(OH)_*n*_^3^ ^– *n*^ + H^+^. The average OH^–^ ligand number coordinated to Nd^3+^ was found to increase from 1.0 to 1.6 at 25 °C and from 1.2 to 2.8 at 75 °C over a pH range from 6.3 to 9.0. The measured speciation shows an increased predominance of Nd­(OH)_3_^0^ over the Nd­(OH)_2_^+^ and Nd­(OH)^2+^ species with increased temperature and pH. The increasing cumulative formation constants (log*β°_*n*_, *n* = 1 to 3) retrieved from 25 to 75 °C differ by 0.1 to 1.9 logarithmic units in comparison to existing thermodynamic databases. These updated thermodynamic data have important implications for geochemical modeling of the speciation of Nd hydroxyl complexes and the solubility of monazite as a function of pH.

## Graphical abstract

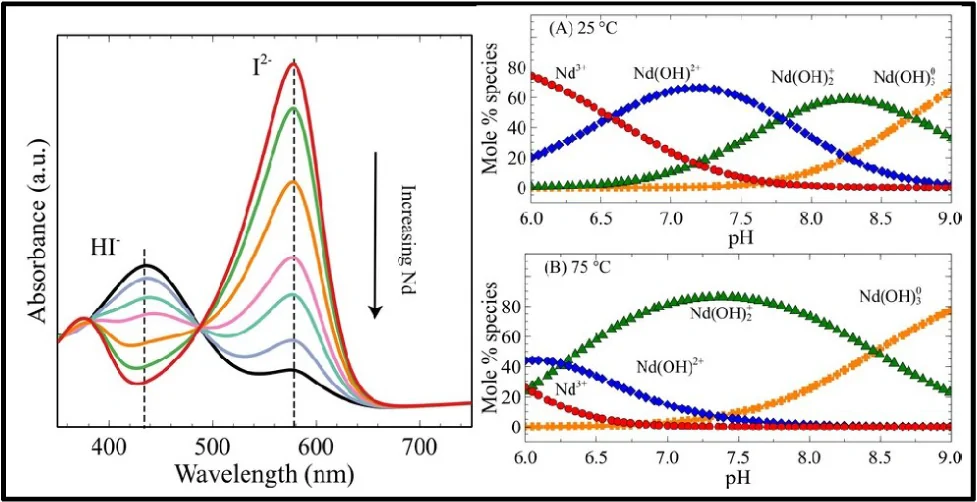

## 1 Introduction

Rare earth elements (REE) are classified as critical minerals used in a wide range of green energy and high-tech applications.^1^ They comprise the lanthanides (La–Lu), yttrium (Y), and scandium (Sc) and have been further subdivided into light and heavy REE based on their chemical properties (e.g., atomic mass and ionic radius).^2,3^ Neodymium (Nd) is primarily used in the production of high-tech and clean-energy equipment because of its magnetic properties.^4,5^ The increased demand for Nd has resulted in interest in its behavior under hydrothermal conditions in natural geological systems and in chemical processes for its separation and recovery. The speciation of Nd in aqueous solution controls its mobilization and fractionation in hydrothermal waters.^6–8^ Accurate determination of the thermodynamic properties of aqueous species is important for understanding the behavior of Nd in aqueous fluids and determining the solubility of REE minerals such as monazite.^9–12^ Thermodynamics of the aqueous species can also be integrated with kinetics to model REE behavior in natural processes.^13,14^ Under near-neutral to alkaline pH conditions, geochemical models predict that Nd occurs predominantly as a hydroxyl species in aqueous fluids.

A few experimental studies have reported the hydrolysis constants for Nd, but the majority of these investigations were conducted at ambient temperature. For example, Guillaumont et al.^15^ carried out solvent extraction experiments to derive the first hydrolysis constant of Nd in 0.1 mol/L HClO_4_/LiClO_4_ solutions at 25 °C. Stepanchikova et al.^16,17^ conducted UV–vis spectroscopy experiments using the pH color indicator *m*-cresol purple to determine the first to third hydrolysis constants of Nd at 25 °C. Yakubovich and Alekseev^18^ reported the first and third hydrolysis constants of Nd, determined by potentiometric experiments in 0.1 mol/L KNO_3_ solutions at 25 °C. There are also a few studies focused on the Nd aqueous species at elevated temperatures. Wood et al.^19^ performed solubility experiments to determine the first to third hydrolysis constants of Nd in 0.03 and 0.1 mol/kg sodium triflate solutions from 30 to 290 °C. Klungness and Byrne^20^ reported the first hydrolysis constants of Nd in 0.7 mol/L NaClO_4_ solutions from 25 to 55 °C, determined by UV–vis spectrophotometry with the pH color indicator *m*-cresol purple. Han and Gysi^21^ have further developed this method to determine the hydrolysis constants of erbium and lanthanum from 25 to 75 °C.^21,22,23^

In this study, a series of UV–vis spectrophotometric experiments were conducted to investigate the hydrolysis of Nd using the color indicator *m*-cresol purple at near-neutral to alkaline pH from 25 to 75 °C at low ionic strength (≤ 0.001 mol/kg). Hydrolysis constants for the Nd­(OH)^2+^, Nd­(OH)_2_^+^, and Nd­(OH)_3_^0^ species were derived and are compared with values reported in the literature. The updated constants have been incorporated into the MINES thermodynamic database,^24^ facilitating geochemical modeling of Nd speciation and monazite solubility at hydrothermal conditions.

## 2 Material and Methods

### 2.1 Materials

Experimental *m*-cresol purple (*m*CP), NdCl_3_, and NaOH-bearing starting solutions were prepared by dilution of three stock solutions to achieve final concentrations of 0.022 mmol/kg *m*CP, 0.334 mmol/kg NaOH, and varying initial concentrations of NdCl_3_ ranging from 0 to 0.155 mmol/kg. The *m*CP stock solution (0.15 mmol/kg) was prepared by dissolving high-purity *m*CP powder (Acros Organics, indicator grade) in Milli-Q water (18.2 MΩ·cm). The NaOH stock solution (0.005 mol/kg) was prepared by diluting NIST-traceable standard NaOH (Inorganic Ventures, 0.9968 ± 0.001 M) with Milli-Q water. The Nd chloride stock solution was prepared by dissolving NdCl_3_·6H_2_O (Sigma-Aldrich, ≥ 99.9% purity) into Milli-Q water, and the Nd concentration was verified by ICP-OES. The pH of the experimental Nd-bearing and Nd-free *m*CP-NaOH solutions ranges from ∼6 to 10, controlled by the initial NaOH concentration and the addition of NdCl_3_ due to the hydrolysis of Nd. All solutions were freshly prepared each day using CO_2_-degassed and ion-exchange column-purified Milli-Q water to minimize any interference from dissolved atmospheric CO_2_.

### 2.2 Analytical Method

Neodymium concentrations in the stock Nd chloride solutions were measured using an Agilent 5900 inductively coupled plasma optical emission spectroscopy (ICP-OES) at the Analytical Chemistry Laboratory of the New Mexico Bureau of Geology and Mineral Resources, New Mexico Institute of Mining and Technology. A 1000 ppm indium stock solution (Inorganic Ventures, trace metal grade) was diluted to 10 ppm in 2% HNO_3_ and used as an internal standard to correct for instrumental drift during analysis. Calibration standards for Nd, ranging from 25 to 250 ppb, were prepared by diluting a 10 ppm multi-REE standard solution (Inorganic Ventures, NIST traceable) in a 2% HNO_3_ blank matrix (Fisher Scientific, trace metal grade). The analytical precision for Nd measurements in the diluted stock solutions (1.45–2.17 ppm) was better than 1.5%. The ICP-OES instrument limit of detection is 2.5 ppb for Nd and was determined from repeated measurements of the procedural blanks (5σ).^25^

The pH of Nd-free *m*CP-NaOH solution was measured at temperatures between 25 and 75 °C using a Metrohm combined pH electrode (serial number 6.0260.010) and a Metrohm 913 pH meter (). The calibration of this combined pH electrode was carried out for each isotherm using Fisher Scientific commercial buffer solutions (pH values of 4, 7, and 10, with an accuracy of 0.01 pH units at 25 °C) with known pH values as a function of temperature. Temperature control of these samples and buffer solutions was made within ± 0.5 °C by submerging the solution vials into a water bath.

### 2.3 In Situ pH Measurements by UV–vis Spectrophotometry

*In situ* pH and Nd hydrolysis were determined in the Nd-free and Nd-bearing *m*CP-NaOH solutions using UV–vis spectrophotometry at temperatures of 25–75 °C. The method used in this study is similar to that of Han and Gysi.^21^ Temperature was controlled and maintained using a Peltier-cell sample holder equipped with a temperature probe, accurate to within ± 0.1 °C of the determined value. The UV–vis spectrophotometric measurements were performed using an Agilent Cary 5000 dual-beam spectrophotometer with a 2.0 nm slit bandwidth. The absorbance spectra of *m*CP at wavelengths ranging from 350 to 750 nm were collected in each experimental *m*CP-NaOH solutions, containing varying initial Nd concentrations from 0 to ∼0.155 mmol/kg at low ionic strength (≤0.001 mol/kg). The absorbance intensities of the deprotonated (I^2–^) and protonated (HI^–^) forms of *m*CP were collected at their maximum absorbance wavelengths, 578 nm and 435 to 428 nm, respectively. The experimental solutions were sealed in 10 mm quartz cuvettes during analysis. The pH of these measured solutions ranges from 6 to 10 (Table S1). Absorbance spectra of *m*CP were measured in at least triplicate series covering the entire pH range for each isotherm. The Nd-bearing *m*CP-NaOH solutions with the highest Nd concentrations (∼0.155 mmol/kg) display a transparent yellow color controlled by the protonated form of the *m*CP chromophore.

## 3 Data Treatment

### 3.1 In Situ pH Measurement in the Experimental Solutions

The pH values in the Nd-free and Nd-bearing *m*CP-NaOH solutions were determined based on the absorbance intensity ratio of the deprotonated (I^2–^) and protonated (HI^–^) forms of *m*CP, which were measured using UV–vis spectrophotometry as follows:

R1 $${\mathrm{HI}}^{-}↔I^{\mathrm{2-}}{+H}^{+}$$

The absorbance intensity of I^2–^ and HI^–^ species is related to pH, which is controlled by the initial concentrations of *m*CP and NaOH and the amount of Nd added to the experimental solutions. The addition of NdCl_3_ lowers the pH of the initially alkaline solutions because of the hydrolysis reaction of Nd^3+^ and the release of protons (H^+^) from water according to

$${\mathrm{Nd}}^{3+}{+nH}_{2}O↔\mathrm{Nd}{\left(\mathrm{OH}\right)}_{n}^{3-n}{+nH}^{+}$$ R2

This pH decrease drives the *m*CP dissociation equilibrium toward the protonated form (HI^–^). As a result, the UV–vis absorbance of the protonated HI^–^ species increases, while the absorbance associated with the deprotonated I^2–^ species correspondingly decreases. Therefore, the solution pH is determined *in situ* based on the spectrophotometric characteristics of *m*CP, including the absorbance intensity ratio (*R*) of I^2–^ and HI^–^ (defined by Abs_I2–_/Abs_HI–_) at their maximum absorbance wavelengths, the molar absorbance coefficients (*e*_1_, *e*_2_, and *e*_3_) of *m*CP, and the dissociation constant (*K* ^o^_*m*CP,T_) of *m*CP at temperature *T* (in °C), using the following equation:

$${\mathrm{pH}}_{T}=-\log e_{2}K_{mCP,T}^{o}+\log\left(\frac{R-e_{1}}{1-R\frac{e_{3}}{e_{2}}}\right)$$ 1

The temperature-dependent optical properties (*e*_1_ to *e*_3_) and dissociation constants of *m*CP were obtained up to 75 °C in the study by Han and Gysi.^21^

### 3.2 Average OH^–^ Ligand Number and Nd Hydrolysis Constant Determination

The average number of OH^–^ (average ligand number, *n⃗*) bound to Nd^3+^ in the Nd-bearing *m*CP-NaOH solutions is calculated based on the measured pH and the known initial OH^–^ concentrations from the NaOH addition. The value of *n⃗* represents the mean number of OH^–^ bound to Nd^3+^ by averaging the contributions of all hydroxyl species (Nd­(OH)_*n*_^3 – *n*^) using the following equation:

$$\mathrm{n⃗}=\frac{\sum {nm}_{\mathrm{Nd}{\left(OH\right)}_{n^{3-n}}}}{m_{\mathrm{Nd}}}=\frac{m_{{\mathrm{OH}}^{-}}-{m^{\prime }}_{{\mathrm{OH}}^{-}}}{m_{\mathrm{Nd}}}$$ 2

where *m*_OH_^–^ and *m′*_OH_^–^ correspond to the amount of OH^–^ in the absence and presence of Nd in the *m*CP-NaOH solutions, respectively. The OH^–^ concentrations in the solutions depend on the initial NaOH added, the ionization constant of water (K_w_) with temperature, and the hydrolysis of Nd. $\sum {nm}_{\mathrm{Nd}{\left(\mathrm{OH}\right)}_{n}^{3-n}}$ corresponds to the total number of OH^–^ bound to Nd^3+^ in the Nd-bearing *m*CP-NaOH solution. The latter can be calculated from the difference of the OH^–^ number between the absence and presence of Nd in the *m*CP-NaOH solutions; m_Nd_ corresponds to the molality of total dissolved Nd in solutions.

The Nd hydrolysis constants (log*β_*n*_^°^, *n* = 1 to 3) for Reaction 2 can be expressed as follows:

βn0*=mNd(OH)n3−nmH+nmNd3+γNd(OH)n3−nγH+nγNd3+ 3

where m_H^+^_, m_Nd^3+^_, and *m*_Nd(OH)_*n*_^3–*n*^_ are the molality concentrations of the respective species, and γ_H^+^_, γ_Nd^3+^_, and γ_Nd(OH)_*n*_^3–*n*^_ are their activity coefficients. Due to the low ionic strength of solutions (≤0.001 mol/kg), the activity coefficients in eq 5 were assumed to be unity. The values of log*β_*n*_^°^ (*n* = 1 to 3) can be determined for every isotherm from the average ligand number and the *in situ* measured pH of the experimental solutions through a combination of eqs 4 and 5 as follows:

n⃗=(1−n⃗)×β*10mH++(2−n⃗)×β*20mH+2+(3−n⃗)×β*30mH+3 4

The cumulative Nd hydrolysis constants are determined by applying linear regression to the normal equation (P = inv­(X′ × X) × (X′ × Y)) using MATLAB (Version R2023a). For each isotherm, the uncertainty of the cumulative hydrolysis constants was determined by calculating the standard deviation of the mean of a triplicated to quadruplicated series (Table S1) of experiments conducted over the pH range of 6.3–10.2.

## 4 Results and Discussion

### 4.1 UV–vis Absorbance as a Function of Nd Concentration at 25–75 °C

The absorbance spectra of the I^2–^ and HI^–^ species were recorded from 25 to 75 °C in the *m*CP-NaOH solutions containing varying initial Nd concentrations ranging from 0 to ∼0.155 mmol/kg (Figure 1 and Table S1). In the Nd-free *m*CP-NaOH solution at 25 °C, the I^2–^ species (depronated form of *m*CP) exhibits a strong absorbance peak at 578 nm, while the HI^–^ species (protonated form of *m*CP) displays a broad valley at 435 nm (Figure 1). Because of the temperature dependence of pi-stacking of the *m*CP molecules and water ionization (*K*_w_), the absorbance spectra of HI^–^ species of *m*CP shifts slightly from 435 to 428 nm with increasing temperature. These results are consistent with the UV–vis data for *m*CP over the temperature range of 25–75 °C reported by Han and Gysi.^21,22^ In the Nd-bearing *m*CP-NaOH solution at 25 °C, the absorbance intensity of I^2–^ species at 578 nm gradually decreases, accompanied by a gradual increase in the absorbance intensity of the HI^–^ species at 435 nm with the addition of up to ∼0.155 mmol/kg Nd. Furthermore, the UV–vis spectra for the HI^–^ species in *m*CP-NaOH solutions with Nd concentrations above 0.122 mmol/kg exhibit a distinct UV–vis absorbance peak. A well-defined isosbestic point is observed at 488 nm between the I^2–^ and HI^–^ species, indicating a constant *m*CP concentration in the NaOH solutions regardless of the presence or absence of Nd^3+^. This indicates that no significant complexation occurred between *m*CP and Nd under the studied experimental conditions. These results are consistent with the study by Schwarzenbach and Flaschka, which indicates that *m*CP does not form complexes with metal ions.^26^ The isosbestic point between I^2–^ and HI^–^ species displays a blue shift from 488 to 483 nm when the temperature is increased from 25 to 75 °C, which aligns with the results published by Liu et al.^27^

**Figure 1:**
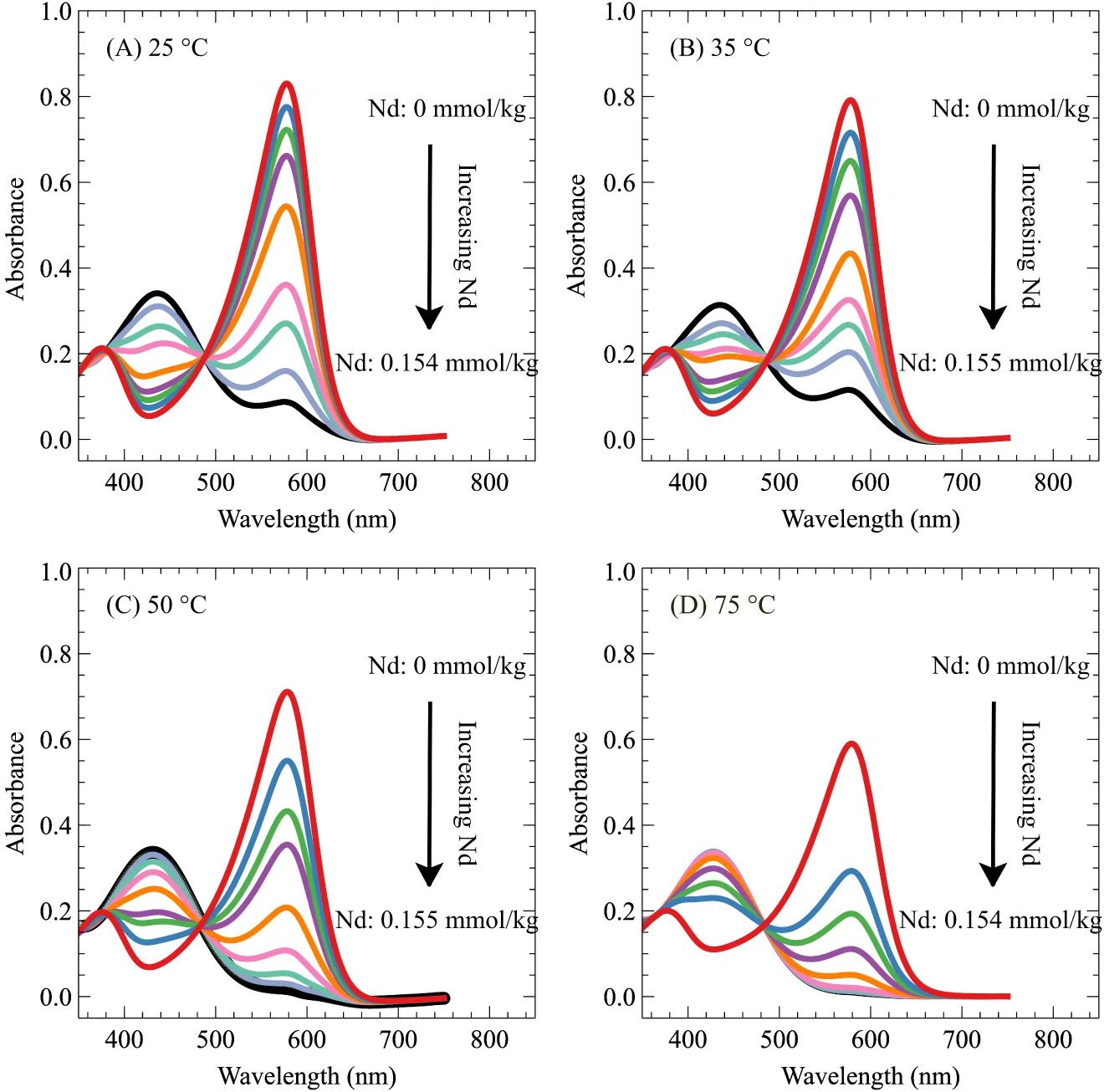
Representative UV–vis spectra of 0.022 mmol/kg *m*CP in 0.334 mmol/kg NaOH with the addition of varying Nd concentrations ranging from 0.000 to 0.155 mmol/kg at (A) 25 °C, (B) 35 °C, (C) 50 °C, and (D) 75 °C. The Nd concentration in each solution and the calculated absorbance intensity ratio (*R*, defined by Abs_I2–_/Abs_HI–_) are listed in Table S1.

The absorbance intensity ratio (*R*) of the I^2–^ to the HI^–^ species for the *m*CP, expressed as Abs_I2–_/Abs_HI–_, was determined from the UV–vis measurements of the Nd-free and Nd-bearing *m*CP-NaOH solutions (Figure 2 and Table S1). As shown in Figure 2, the *R* value decreases with increasing concentrations of Nd and temperatures. At 25 °C, the *R* value decreases from ∼14.56 to 0.44 as the Nd concentration increases from 0 to ∼0.155 mmol/kg. At elevated temperatures of 35, 50, and 75 °C, the *R* values decrease from ∼13.01 to 0.35, ∼11.11 to 0.06, and ∼5.37 to 0.03, respectively. These results indicate significant changes in the relative concentrations of I^2–^ and HI^–^ species with both Nd concentration and temperature. The corresponding pH values of these Nd-free and Nd-bearing *m*CP-NaOH solutions were inferred based on the observed spectral changes.

**Figure 2:**
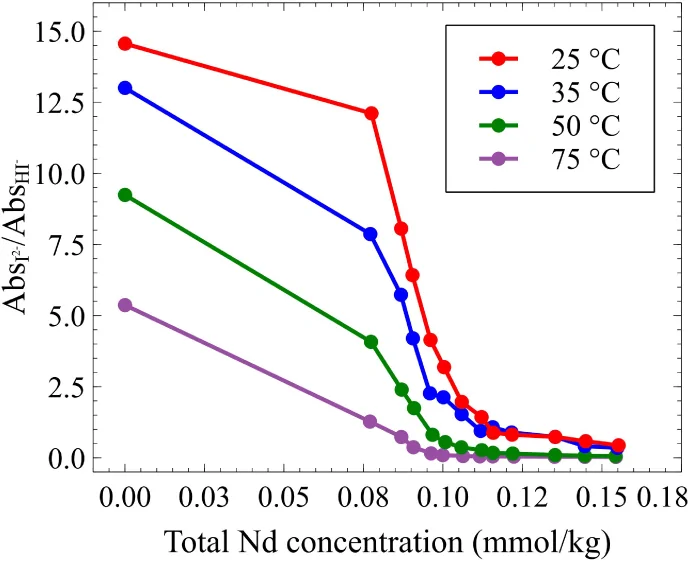
Absorbance intensity ratio (*R,* Abs_I2–_/Abs_HI–_) of I^2–^ to HI^–^ at their maximum wavelengths with the addition of different concentrations of Nd ranging from 0.000 to ∼0.155 mmol/kg at temperatures up to 75 °C. The experimental data are listed in Table S1.

### 4.2 pH Determination from UV–vis Measurements

The pH values of these Nd-free and Nd-bearing *m*CP-NaOH solutions were obtained from the derived absorbance intensity ratio of the I^2–^ to the HI^–^ species and the optical properties and dissociation constant of *m*CP at each temperature (eq 3). The pH values are listed in Table S1 and shown in Figure 3. Polynomial fits using the equation pH= a + bx^1/2^ + cx^–2^ as a function of total Nd concentration at each isotherm with their regressed parameters and coefficients are listed in Table 1. The pH values of these Nd-free and Nd-bearing *m*CP-NaOH solutions decrease with increasing Nd concentrations and temperature. In the Nd-free *m*CP-NaOH solutions, the pH values are ∼10.2, 9.9, 9.5, and 9.1 at 25, 35, 50, and 75 °C, respectively. In the presence of varying concentrations of Nd in the *m*CP-NaOH solutions, the pH values of these solutions significantly decrease. At 25 °C, increasing the Nd concentration from ∼0.1 to 0.155 mmol/kg results in a decrease in the pH value of *m*CP-NaOH solutions from ∼8.9 to 7.9. This trend becomes more significant at elevated temperatures. At 35 °C, the pH of *m*CP-NaOH solutions decreases from ∼8.7 to 7.7 with increasing Nd concentration from ∼0.1 to 0.155 mmol/kg. At 50 and 75 °C, the pH of *m*CP-NaOH solutions decreases from ∼8.6 to 6.7 and from ∼8.3 to 6.3, respectively. These results indicate that the initial pH for given NaOH solution is different at each temperature due to K_w_. However, the decrease in pH due to the addition of Nd is steeper at 75 °C over lower temperature (Figure 3), indicating more significant hydrolysis of Nd and higher coordination of OH^–^ to Nd^3+^. Therefore, the pH value of Nd-bearing *m*CP-NaOH solutions at each temperature depends on the addition of Nd concentration due to the enhanced hydrolysis and consequent release of protons that occurs when free Nd^3+^ is introduced into the *m*CP-NaOH solutions (Reaction 2).

**Table 1:** Relationship between the pH Values and Varying Concentrations of Nd at 25–75 °C and Their Regressed Fitting Parameters and Coefficients^a^

	Fitted equation = a + bx^1/2^ + cx^–2^				
*T* (°C)	a	b	c	R^2^	RMSE
25	4.240	6.649	0.024	0.902	0.113
35	5.684	3.756	0.014	0.771	0.155
50	5.483	1.454	0.019	0.945	0.119
75	0.959	10.541	0.029	0.903	0.197

a These experimental data are listed in Table S1.

**Figure 3:**
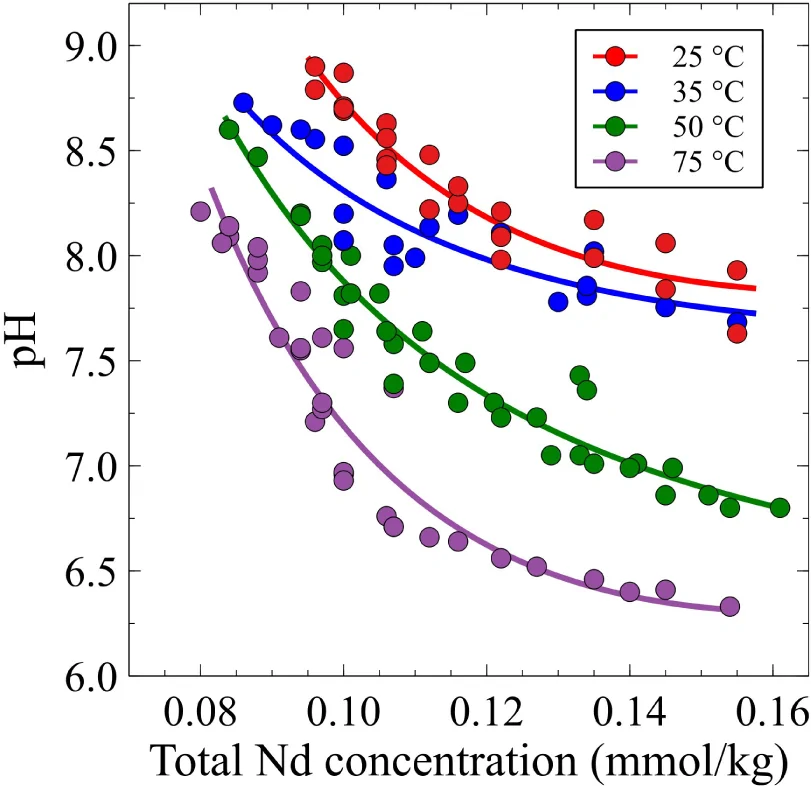
pH values of 0.022 mmol/kg *m*CP in 0.334 mmol/kg NaOH with the addition of varying Nd concentrations ranging from ∼0.08 to 0.155 mmol/kg using UV–vis spectrophotometry at 25, 35, 50, and 75 °C. Also shown are the fits to the experimental data. The experimental data, fitted equations, and coefficients are listed in Table 1 and Table S1.

### 4.3 Average OH^–^ Ligand Number as a Function of pH at 25–75 °C

The average number of OH^–^ ligands (Table S1) bound to Nd^3+^ in the Nd-bearing *m*CP-NaOH solutions is determined through pH measurements by using UV–vis at 25–75 °C (eq 4). Figure 4 shows the calculated average ligand number as a function of pH at each temperature and their polynomial fits y= a + bx + cx^2^. The regression parameters and coefficients of these fits (R^2^ and RMSE) are listed in Table 2. Because the initial OH^–^ concentration was kept constant at each isothermal condition, as the NdCl_3_ concentration increases, the hydrolysis of Nd results in a decrease in the average OH^–^ ligand number coordinated to Nd^3+^ (eq 4). This is consistent with the results of *in situ* pH measurements in this study (Figure 3). At 25 °C, the average ligand number coordinated to Nd^3+^ increases from ∼1.0 at pH 7.6 to ∼1.6 at pH 8.9, indicating that Nd­(OH)^2+^ is the dominant species at pH below 7.6, with a gradual transition toward Nd­(OH)_2_^+^ species between pH 7.6 and 8.9. A similar trend is observed at elevated temperatures. The average ligand number increases from ∼1.2 to 1.8 over the pH of 7.6–8.8 at 35 °C, whereas at 50 °C, the average ligand number ranges from ∼1.2 to 2 at pH between 6.6 and 8.6. These observations suggest that Nd­(OH)^2+^ and Nd­(OH)_2_^+^ are the predominant species at 25–50 °C within a pH range of 7.6–8.6. At 75 °C, the average ligand number increases from ∼1.4 to 2.7 at pH between 6.3 and 8.1, indicating an increase in the predominance of the Nd­(OH)_3_^0^. Across all temperatures (25–75 °C) and pH values (6.3–8.6), the average ligand number remains below 3, suggesting that the formation of Nd­(OH)_4_^–^ species does not occur under the studied experimental conditions. This finding is consistent with previous studies,^19,22,23,28^ which report the absence of such REE­(OH)_4_^–^ species in alkaline solutions.

**Table 2:** Relationship between the Nd Average Ligand Number and the Measured pH Values at 25–75 °C and Their Regressed Parameters and Coefficients^a^

	Fitted equation = a + bx + cx^2^				
*T* (°C)	a	b	c	R^2^	RMSE
25	–16.662	3.935	–0.211	0.757	0.088
35	–44.040	10.634	–0.617	0.649	0.128
50	–10.306	2.770	–0.154	0.820	0.101
75	–29.857	8.281	–0.527	0.836	0.191

a The full dataset is listed in Table S1.

**Figure 4:**
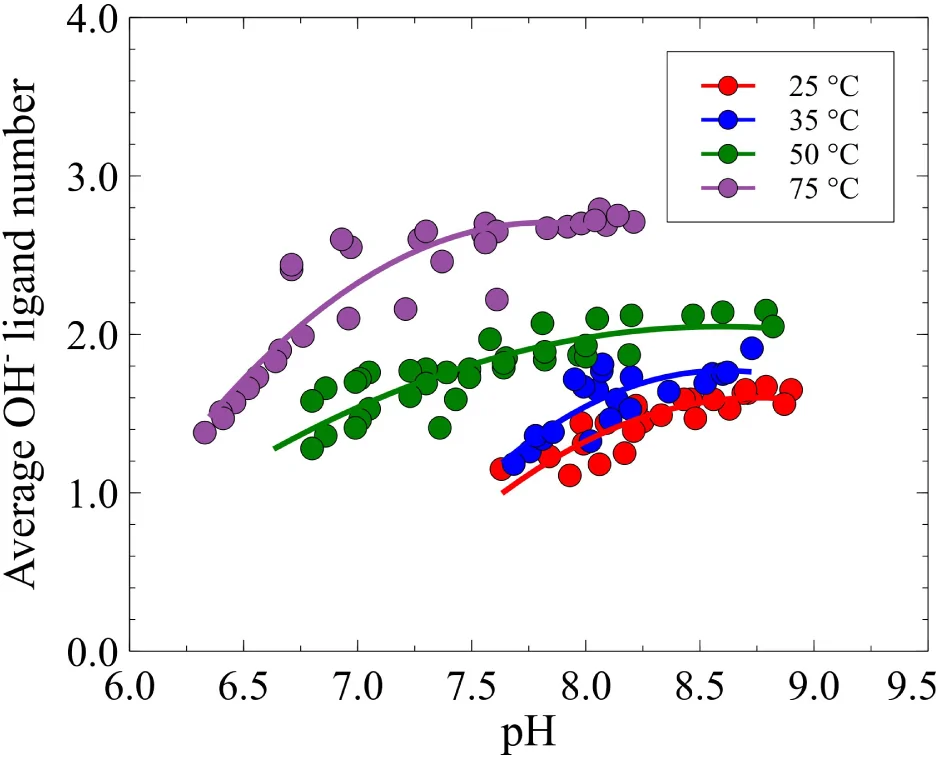
Average number of OH^–^ ligands bound to Nd^3+^ and the corresponding determined pH values in the Nd-bearing *m*CP-NaOH solutions by using UV–vis at temperatures ranging from 25 to 75 °C. Also shown are the fits to the experimental data. The experimental data, fitted equations, and coefficients are listed in Table S1 and Table 2.

Only a limited number of experimental studies have reported the Nd hydroxyl complexes distribution as a function of pH. Stepanchikova and Biteikina^16^ determined the Nd hydrolysis constants at 25 °C by using *m*CP over the pH range of 6.1–8.6 and 2-naphthol at pH of 9–9.8. Their results indicate that Nd­(OH)^2+^, Nd­(OH)_2_^+^, and Nd­(OH)_3_^0^species are the predominant species at pH ∼ 9, which is consistent with the Nd average ligand numbers derived in this study at 25 °. Wood et al.^19^ conducted a series of solubility studies on solid Nd­(OH)_3_ to investigate aqueous Nd hydroxyl species over a temperature range of 30–290 °C in 0.03 mol/kg sodium triflate solutions. Their results indicate that Nd^3+^ and Nd­(OH)_3_ **^0^** species are predominant and control hydrolysis at temperatures between 30 and 100 °C and pH between 5.2 and 10. At 250 and 290 °C, their study indicates that Nd**^3+^**, Nd­(OH)_2_ **^+^**, and Nd­(OH)_3_ **^0^** species are present in solutions with pH above 3.2. Additionally, the presence of Nd­(OH)_2_ **^+^** species at 290 °C appears to be sensitive to ionic strength, suggesting its stability may be influenced by the experimental conditions (e.g., ionic strength and high temperature).

### 4.4 Determination of Cumulative Nd Hydrolysis Constants (*β_*n*_^°^) at 25–75 °C

The cumulative Nd hydrolysis constants were determined based on the calculated average ligand number and the measured pH values in the Nd-bearing*m*CP-NaOH solutions using eq 6. The logarithmic values of the cumulative hydrolysis constants (log*β_1_^°^ to log*β_3_^°^), corresponding to the formation of Nd­(OH)^2+^, Nd­(OH)_2_^+^, and Nd­(OH)_3_^0^ species, are presented in Table 3. These values are compared with available literature data and theoretical predictions over the temperature range of 25–75 °C in Figure 5. The values of log*β_*n*_^°^(*n* = 1 to 3) for the Nd­(OH)^2+^, Nd­(OH)_2_^+^, and Nd­(OH)_3_^0^ species derived in this study were fit as a function of temperatures between 25 and 75 °C by using the equation log*β_*n*_^°^ = a + bx + c/x, where x is the temperature in kelvin (Table 4). A comparison of the cumulative hydrolysis constants for Nd­(OH)^2+^, Nd­(OH)_2_^+^, and Nd­(OH)_3_^0^species indicates that the stability of these species does not display significant differences at temperatures between 25 and 35 °C (Figure 5). However, the cumulative hydrolysis constants increase significantly from 25 to 75 °C, i.e., log*β_1_^°^ increases by about 1 order of magnitude, whereas the log*β_2_^°^ and log*β_3_^°^ increase by ∼2 orders of magnitude (Table 3).

**Table 3:** Logarithmic Values of Cumulative Nd Hydrolysis Constant (log*β_*n*_^°^, *n* = 1 to 3) Determination for the Reaction Nd^3+^ + *n*H_2_O = Nd­(OH)_*n*_^3^ ^– *n*^ + *n*H^+^ at Temperature 25–75 °C by Using UV–vis Spectrophotometry and *m*CP^a^

*T* (°C)	log*β_1_^°^	1σ	log*β_2_^°^	1σ	log*β_3_^°^	1σ
25	–8.1	0.2	–15.8	0.2	–24.5	0.1
35	–7.7	0.2	–15.4	0.2	–24.2	0.2
50	–7.3	0.1	–14.6	0.1	–23.1	0.2
75	–6.7	0.1	–13.7	0.2	–22.1	0.1

a The Standard deviation of the mean (1σ) is based on replicate experiments in Table S1.

**Table 4:** Regressed Coefficients for the Logarithm Values (log*β_*n*_^°^, *n* = 1 to 3) of the Cumulative Nd Hydrolysis Constants (Nd^3+^ + *n*H_2_O = Nd­(OH)_*n*_^3^ ^– *n*^ + nH^+^, *n* = 1 to 3) as a Function of Temperature from 25 to 75 °C^a^

	a	b	c	R^*2*^	RMSE
β_1_	29.180	–7.249	–43.396	1.000	0.013
β_2_	29.852	–9.354	–47.995	0.997	0.084
β_3_	–26.920	–2.014	30.670	0.988	0.208

a These regressions are generated using the experimental data from Table 3. The regressed function used is log*β*_n_* ^°^ = a + bx + c/x, where x is the temperature in kelvin.

**Figure 5:**
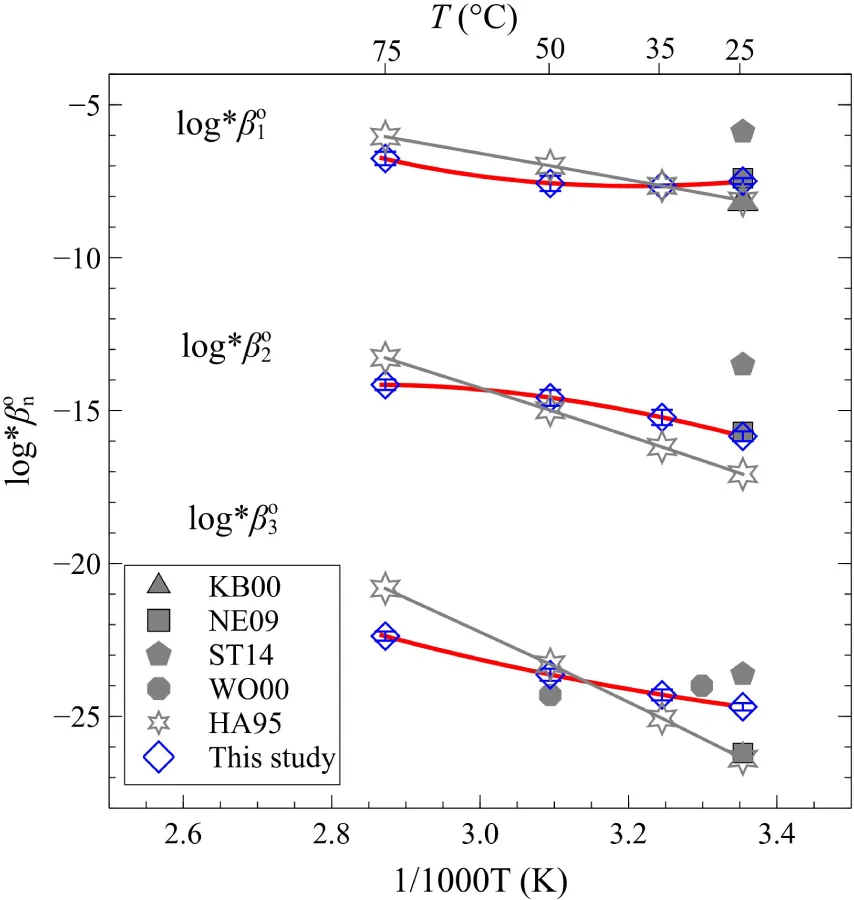
Cumulative hydrolysis constant (log*β_*n*_^°^, *n* = 1 to 3) of Nd (REE^3+^ + *n*H_2_O = REE­(OH)_*n*_^3 – *n*^ + *n*H^+^, with *n* = 1 to 3) as a function of temperature between 25 and 75 °C showing a comparison of the experimental data from this study (Table 3) with the extrapolations from Haas et al.^29^ The regressed coefficients for fits to our data are listed in Table 5. Additional references include (Table 5): [ST14];^17^ [KB00];^20^ [NE09];^30^ [WO00];^19^ [HA95].^29^

There are few experimental studies that have investigated Nd hydrolysis at temperatures of 25–290 °C (Figure 5 and Table 5). Most of these experimental studies were performed at 25 °C. Stepanchikova et al.^17^ conducted UV–vis experiments and determined the log*β_n_^°^ (*n* = 1 to 3) values for the hydrolysis of Nd at 25 °C, with values of −5.9, −13.5, and −23.6, respectively. Neck et al.^30^ performed solubility experiments for solid Nd­(OH)_3_ and derived log*β_1_^°^ and log*β_2_^°^ values of −7.5 and −15.7, respectively. Klungness and Byrne^20^ carried out potentiometric experiments in 0.7 mol/L NaClO_4_ solutions and derived log*β_1_^°^ values of −8.2, −7.9, −7.5, and −7.4 at 25, 35, 50, and 55 °C, respectively. Wood et al.^19^ performed solubility experiments in 0.03 and 0.1 mol/kg sodium triflate solutions to investigate Nd hydrolysis from 30 to 290 °C. Their log*β_1_^°^ values are −3.8 and −3.3 at 250 and 290 °C, their log*β_2_^°^ value is −8.4 at 290 °C, and their log*β_3_^°^ values are −24.0, −24.3, −22.1, and −13.9 at 30, 50, 100, and 290 °C, respectively. Since most of these experimental data available in the literature were obtained either only at 25 °C or at high ionic strength conditions (Table 5), a comparison with hydrolysis constants above 25 °C is only possible using the extrapolated values from the study by Haas et al.^29^ The thermodynamic properties of Nd hydroxyl complexes in this latter study are predicted at high temperature and pressure from the Helgeson–Kirkham–Flowers (HKF) equation of state parameters based on few experimental data at low temperature.^31–33^ The cumulative hydrolysis constants derived in this experimental study differ from those predicted by Haas et al.^29^ by approximately 0.1 to 1.9 log units (Table 6). Further comparison of the temperature dependence of cumulative Nd hydrolysis constants indicates that the log*β_1_^°^ values obtained in this study between 25 and 50 °C are consistent with those reported by Haas et al.,^29^ whereas a discrepancy is observed at 75 °C. However, the log*β_2_^°^ and log*β_3_^°^ derived from our study differ notably from the predicted values. Specifically, in the relatively lower temperature range (25–50 °C), the predictions tend to underestimate the stability of the Nd­(OH)_2_^+^ and Nd­(OH)_3_^0^ species, whereas in the higher temperature range (50–75 °C), they tend to overestimate it.

**Table 5:** Compilation of Available Literature Data for the Cumulative Nd Hydrolysis Constants log*β_1_ to log*β_3_

Author	Method	*T* (°C)	Ionic strength and medium^a^	log*β_1_ ^b^	log*β_2_ ^b^	log*β_3_ ^b^
Kragten and Decnop-Weever^34^	Solubility	21.5	1 M NaClO_4_	–8.1	–16.2	–24.3
Frolova et al.^35^	Potentiometry	25	3 M NaClO_4_	–9.57		
Baes and Mesmer^36^	Empirical fits and data compilation	25	0.1 M	–8.0		
Guillaumont et al.^15^	Solvent extraction	25	0.1 M HClO_4_/LiClO_4_	–7.0		
Klungness and Byrne^20^	Potentiometry	25	infinite dilution	–8.18		
Yakubovich and Alekseev^18^	Potentiometry	25	0.1 M KNO_3_	–8.24		–25.16
Stepanchikova et al.^17^	Spectrophotometry	25	0.0005 M	–5.88	–13.50	–23.62
Neck et al.^30^	Solubility	25	infinite dilution	–7.4	–15.4	
Wood et al.^19^	Solubility	30	0.03 m, NaTr			–24
Klungness and Byrne^20^	Potentiometry	40	0.7 M NaClO_4_	–8.09		
Wood et al.^19^	Solubility	50	0.03 m, NaTr			–24.3
Klungness and Byrne^20^	Potentiometry	55	0.7 M NaClO_4_	–7.73		
Wood et al.^19^	Solubility	100	0.03 m, NaTr			–22.1
Wood et al.^19^	Solubility	250	0.03 m, NaTr	–3.8		
Wood et al.^19^	Solubility	290	0.1 m, NaTr		–8.4	
Wood et al.^19^	Solubility	290	0.03 m, NaTr	–3.3		–13.9

a Concentration units in M are mol/L; m is mol/kg.

b Note that the symbol (^°^) was omitted because a few studies at high ionic strength report conditional equilibrium constants not extrapolated to infinite dilution.

**Table 6:** Comparison between the Cumulative Nd Hydrolysis Constants Derived from the Fitted Temperature Function from This Experimental Study (Table 3) and the Values Retrieved from the Thermodynamic Property by Haas et al^29^ ^a^

*T* (°C)	log*β_1_° (this study)	log*β_1_^°^ (Haas et al.)^29^	Δ	log*β_2_^°^ (this study)	log*β_2_^°^ (Haas et al.)^29^	Δ	log*β_3_^°^ (this study)	log*β_3_° (Haas et al.)^29^	Δ
25	–8.1	–8.1	0.1	–15.8	–17.1	1.2	–24.5	–26.4	1.9
35	–7.7	–7.7	0.1	–15.3	–16.2	0.9	–24.0	–25.1	1.1
50	–7.3	–7.0	0.3	–14.6	–15.0	0.4	–23.2	–23.3	0.1
75	–6.8	–6.0	0.7	–13.7	–13.3	0.5	–22.0	–20.8	1.2

a Δ represents the differences between our study and Haas et al.^29^

### 4.5 Impact of the Updated Hydrolysis Constants for Modeling the Speciation of Nd as a Function of pH and Temperature

The updated cumulative Nd hydrolysis constants in this study were used to model Nd speciation over a pH range of 6–9 and temperatures from 25 to 75 °C using HCl/NaOH titration simulations in the GEMS program.^37^ Speciation results of Nd hydroxyl species and the Nd^3+^ aqua ion were compared to those generated using thermodynamic properties predicted by Haas et al.^29^ (Figure 6). The thermodynamic properties of Nd hydroxyl species reported by Haas et al.,^29^ along with thermodynamic data for other aqueous species from Shock and Helgeson^38^ and Shock et al.,^39^ are incorporated into the SUPCRT92 program,^40^ which is referred to as the Supcrt92 database.

**Figure 6:**
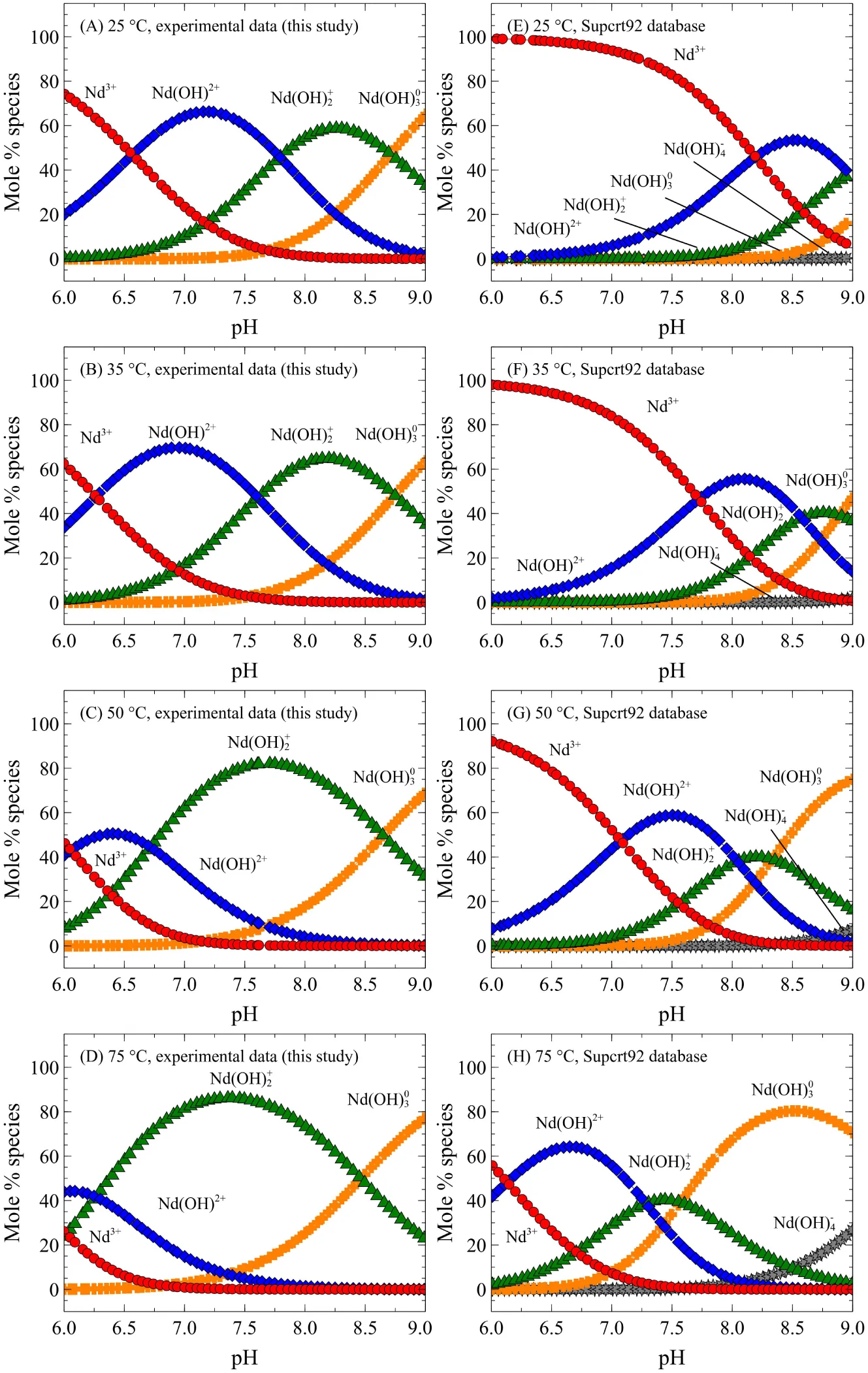
Speciation diagram of the aqueous Nd^3+^ and Nd hydroxyl species at a pH of 6 to 9 and temperatures of 25 to 75 °C using the GEMS code package^37^ and the HCl/NaOH titration model using the cumulative Nd hydrolysis constants derived in this study (A–D) and those predicted using the Supcrt92 database (E–H).

The diagrams of pH and mole % of Nd species calculated from the updated cumulative hydrolysis constants in this study at 25–75 °C and pH of 6–9 are shown in Figure 6A–D. The Nd^3+^ and Nd hydroxyl species shift toward lower pH values as temperature increases. The relative abundance of the Nd­(OH)^2+^ species increases between 25 and 35 °C but decreases at 50 and 75 °C. In contrast, the Nd­(OH)_2_^+^ species exhibits a consistent increase in abundance over the entire temperature range of 25–75 °C. The neutral species of Nd­(OH)_3_^0^ becomes predominant at pH above 8.4–8.7 at temperatures of 25–75 °C. The modeled Nd­(OH)_3_^0^ species stability based on the hydrolysis constants derived from our study is consistent with the potentiometric experiments by Wood et al.,^19^ which indicate that the neutral Nd­(OH)_3_^0^ species predominates above pH ∼ 8.5 at 30–50 °C and above pH ∼ 7.5 at 100 °C.

The diagrams of Nd hydroxyl speciation distribution using the thermodynamic properties of Nd^3+^ and Nd hydroxyl species from the Supcrt92 database are also shown in Figure 6E–H. In the simulations, increasing temperature results in a shift of the distribution of Nd^3+^ and Nd hydroxyl species to acidic pH, which is similar to the experimental results of Nd speciation in this study (Figure 6A–D). In comparison of the Nd hydroxyl speciation distribution at near-neutral to alkaline pH and 25–75 °C, one key difference is the overestimation of the stability of Nd^3+^ and the underestimation of the stability of Nd­(OH)_2_^+^ and Nd­(OH)_3_^0^ species in the model by Haas et al.^29^ For example, at 25 °C, Nd^3+^ is the dominant species at pH below 6.8 in both models. However, our study indicates a significantly higher Nd hydroxyl species stability with a transition at pH of ∼6.8, where Nd­(OH)^2+^ and Nd­(OH)_2_^+^ species become predominant. In contrast, the speciation model based on Haas et al.^29^ predicts that the Nd hydroxyl species only become dominant at pH values above 8.2. Another notable difference is the appearance of the Nd­(OH)_4_^–^ species at pH > 8.6 and 75 °C from the prediction by Haas et al.,^29^ which is not supported by our experimental data. This species was neither observed based on the average ligand number data in this study (Figure 4), nor was it reported in the study of Nd hydrolysis by Wood et al.^19^

### 4.6 Monazite-(Nd) Stability and Aqueous Speciation in Hydrothermal Fluids

Aqueous REE speciation in hydrothermal fluids plays a critical role in the formation and enrichment of REE ore deposits.^6,41,42^ In this study, we carried out geochemical modeling using the GEMS program^37^ and the MINES thermodynamic database^24^ updated with our new cumulative Nd hydrolysis constants (Table 6) to predict the solubility of monazite (NdPO_4_) and Nd mobility in 0.1 and 1.0 mol/L NaCl aqueous solutions at pH of 2–10 and 75 °C (Figure 7). For comparison, we also show the predicted NdPO_4_ solubility using the Supcrt92 database for Nd hydroxyl species. A comparison between our modeling results and those predicted by the Supcrt92 database (Figure 7A) shows that monazite solubility and Nd mobility are approximately 0.5 log m_Nd_ units lower in our model under acidic to near neutral conditions. In the near-neutral to alkaline solutions, both models yield similar results. The solubility difference increases to around 1 log m_Nd_ units in more alkaline conditions. In the simulations from our study, the Nd mobility and monazite solubility at pH below 7 are primarily controlled by the Nd aqueous species such as Nd^3+^, NdCl^2+^, and NdCl_2_^+^. When pH increases to >7.0, the Nd mobility becomes controlled by the hydroxyl species of Nd­(OH)_2_^+^ and Nd­(OH)_3_^0^, and the Nd­(OH)_3_^0^ species becomes predominant at pH > 8.5. Moreover, increasing NaCl concentration from 0.1 to 1.0 mol/L increases the overall solubility of NdPO_4_ at acidic conditions (pH 2.0–5.5), resulting in an overall increase in dissolved Nd concentration of approximately 0.7 log m_Nd_ units. Higher salinity also expands the stability field of Nd chloride species (NdCl^2+^ and NdCl_2_^+^), with their predominance extending up to about pH 7.6 at 75 °C. These findings align with previous experimental^8,13,43^ and modeling studies emphasizing the significant role of Cl-bearing species in enhancing REE transport in saline fluids.

**Figure 7:**
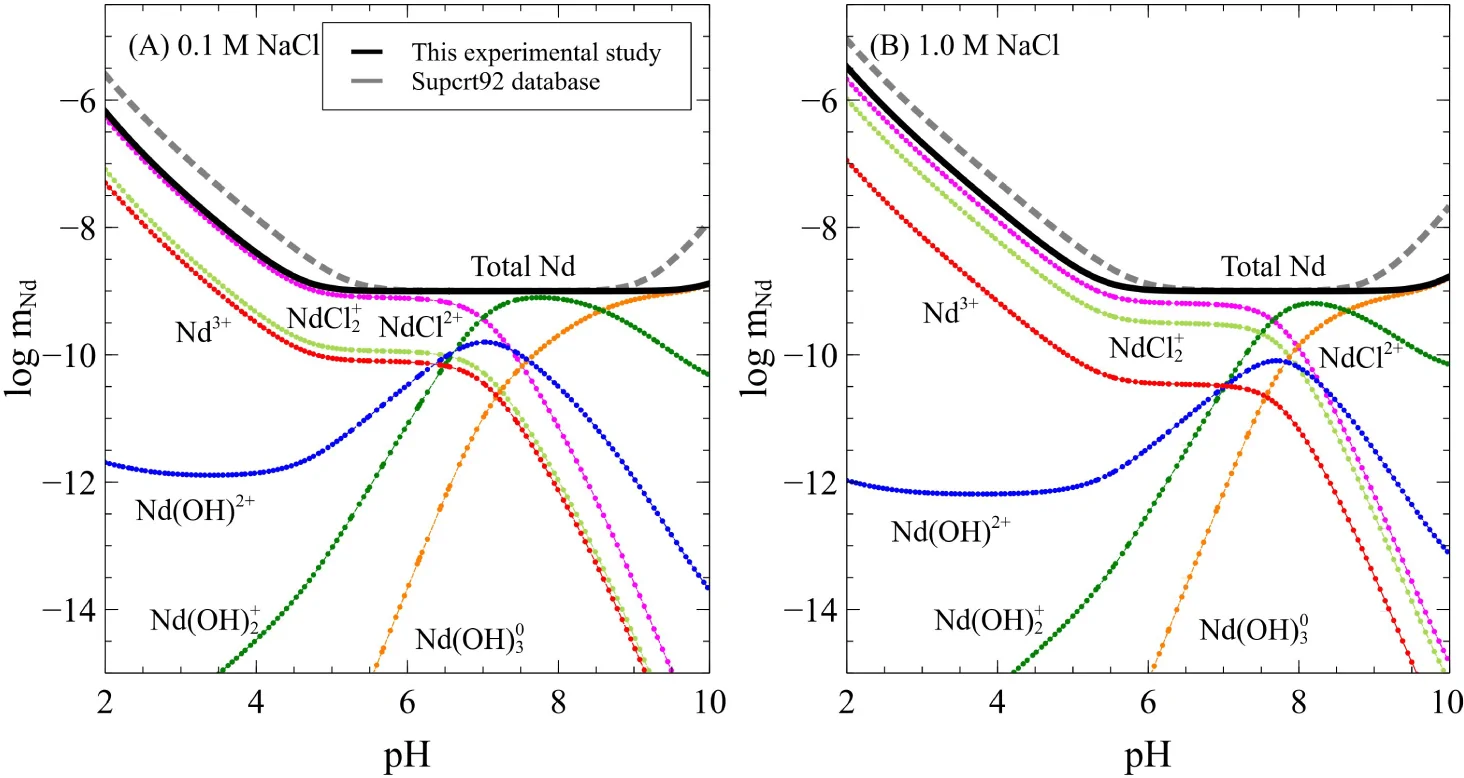
Simulation of monazite (NdPO_4_) solubility and aqueous Nd speciation as functions of pH and salinity at 75 °C on the GEMS program and the MINES thermodynamic database^24^ using the HCl/NaOH titration and the thermodynamic properties of Nd hydroxyl complexes derived in this study. Total Nd concentrations calculated using the Supcrt92 database are shown for comparison. The model assumes the equilibration of NdPO_4_ monazite with aqueous solutions containing 0.1 M NaCl (A) and 1 M NaCl (B). Thermodynamic properties for Nd chloride complexes are from Migdisov et al.^44^ Thermodynamic data for NdPO_4_ and Nd^3+^ are from Van Hoozen et al.^45^ and Pan et al.,^10^ respectively.

## 5 Conclusion

The influence of temperature on the hydrolysis of Nd was investigated using UV–vis spectrophotometry at near-neutral to alkaline pH (6–9). This method enables the simultaneous determination of *in situ* pH and Nd hydrolysis between 25 and 75 °C. The average ligand number and hydrolysis constants derived in our study indicate that the Nd­(OH)^2+^, Nd­(OH)_2_^+^, and Nd­(OH)_3_^0^ species predominate at the studied experimental conditions. Notably, there was no evidence for the presence of Nd­(OH)_4_^–^ species at pH > 9, even at 75 °C. The Nd hydrolysis constants (log*β_1–3_^°^) increase by ∼1–3 log units with temperature from 25 to 75 °C. The temperature-dependent stability of Nd­(OH)_3_^0^ species at alkaline pH is consistent with previous potentiometric results by Wood et al.^19^ However, comparison with the temperature-extrapolated hydrolysis constants from Haas et al.^29^ reveals notable discrepancies. The values of log *β_1_° derived in this study are higher, and both values of log *β_2_° and log* β_3_° depending on temperature are lower than those predicted by Haas et al.^29^ These differences suggest that the thermodynamic properties for Nd hydroxyl complexes by Haas et al.^29^ may overestimate the stability of Nd^3+^ at near-neutral conditions and underestimate the stability of Nd­(OH)_2_^+^ and Nd­(OH)_3_^0^ species at elevated temperatures. Hence, there is a need to revisit and revise the existing temperature functions for the thermodynamic properties of REE hydroxyl complexes. Improved thermodynamic data for the REE hydroxyl complexes will not only strengthen predictive capabilities for REE behavior in geological systems but also broaden the applicability of these models in the development of separation and recovery technologies.

## Supplementary Material





## Data Availability

Data are available through Mendeley Data at DOI: https://doi.org/10.17632/v47j9h6fpg.1.

## Notes

Modeling Nd hydroxyl speciation using the updated constants with the geochemical modeling program GEMS (ZIP)
Brief description of the GEMS geochemical modeling program; UV–vis spectrophotometry-determined absorbance intensity ratios, pH, and average ligand numbers (Table S1) (PDF)
