# Supplementary material for: The Stability of Nd Hydroxyl Complexes at Near Neutral to Alkaline pH from 25 to 75 °C: Implications for Monazite Solubility in Hydrothermal Aqueous Fluids
Source: ACS Earth Space Chem. 2026 Jul 7;10(8):1832–42. doi: 10.1021/acsearthspacechem.5c00328 (PMC13501946; doi:10.1021/acsearthspacechem.5c00328)
Supplement: Supplementary file 1 [file sp5c00328_si_002.pdf]

The Stability of Nd Hydroxyl Complexes at Near Neutral to Alkaline pH from 25 to 75 °C: Implications for Monazite Solubility in Hydrothermal Aqueous Fluids

Hannah J. Han[1]*, Alexander P. Gysi[1,2]

[1]New Mexico Bureau of Geology and Mineral Resources, New Mexico Institute of Mining and Technology, 801 Leroy Place, Socorro, NM 87801, USA

[2]Department of Earth and Environmental Science, New Mexico Institute of Mining and Technology, 801 Leroy Place, Socorro, NM 87801, USA

* Corresponding author: hannah.juan.han@nmt.edu

KEYWORDS: *Rare earth elements, hydrolysis, UV-vis spectrophotometry, pH, REE transport, monazite*

Brief description of GEMS program

The GEMS program (https://gemshub.github.io/site/) is a multi-purpose Gibbs energy minimization code package designed to solves complex multicomponent-multiphase equilibria involving solid solution mixtures, non-ideal aqueous solutions, gas mixtures, supercritical fluids, and sorption phase [37]. GEMS is widely used in geochemical modeling applications, including batch speciation and solubility calculations, solubility and pH titration diagram, and reaction-path and mass-transfer simulations. The GEMS program includes a thermodynamic consistency check for standard state properties, including $\Delta_f G°$, $\Delta_f H°$, and $S°$, at 298.15 K and 1 bar. When all three properties are entered into the thermodynamic database, their internal consistency is evaluated, and any one of the properties can be recalculated from the remaining two. In this study, the MINES thermodynamic database [24] (https://geoinfo.nmt.edu/mines-tdb) was tailored and applied in the combination with the GEMS code package for geochemical modeling.

Table S1. UV-vis spectrophotometric experiments of 0.022 mmol/kg *m*CP in 0.334 mmol/kg NaOH solutions with varying concentrations of Nd, calculated absorbance intensities ratio (R[1]), measured pH (pH_m[2]), and derived average ligand number[3], at temperature of 25 − 75 °C.

Exp	Nd (mmol/kg)	R[1]	pH_m[2]	Average ligand number[3]
T= 25 °C				
Nd-a-0	0.000	14.62	10.20	
Nd-a-1	0.096	3.37	8.90	1.65
Nd-a-2	0.100	2.31	8.71	1.63
Nd-a-3	0.106	1.39	8.46	1.59
Nd-a-4	0.112	0.83	8.22	1.53
Nd-a-5	0.116	0.72	8.16	1.49
Nd-a-6	0.122	0.48	7.98	1.44
Nd-a-7	0.136	0.23	7.65	1.31
Nd-a-8	0.145	0.15	7.45	1.23
Nd-a-9	0.155	0.10	7.26	1.16
Nd-b-0	0.000	14.61	10.20	
Nd-b-1	0.096	2.71	8.79	1.67
Nd-b-2	0.100	2.21	8.69	1.63
Nd-b-3	0.106	1.30	8.43	1.59
Nd-b-4	0.112	0.40	7.90	1.56
Nd-b-5	0.116	0.43	7.93	1.51
Nd-b-6	0.122	0.27	7.72	1.45
Nd-b-7	0.135	0.17	7.51	1.31
Nd-b-8	0.145	0.16	7.48	1.23
Nd-b-9	0.155	0.10	7.25	1.15
Nd-c-0	0.000	14.56	10.19	
Nd-c-1	0.096	4.14	9.02	1.58
Nd-c-2	0.100	3.19	8.87	1.56
Nd-c-3	0.106	1.96	8.63	1.53
Nd-c-4	0.112	1.43	8.48	1.47
Nd-c-5	0.116	0.88	8.25	1.45

Nd-c-6	0.122	0.81	8.21	1.39
Nd-c-7	0.135	0.73	8.17	1.25
Nd-c-8	0.145	0.58	8.06	1.18
Nd-c-9	0.155	0.44	7.93	1.11
Nd-d-0	0.000	14.64	10.20	
Nd-d-1	0.100	2.27	8.70	1.65
Nd-d-2	0.106	1.69	8.56	1.59
Nd-d-3	0.112	0.82	8.22	1.55
Nd-d-4	0.116	1.04	8.33	1.49
Nd-d-5	0.122	0.62	8.09	1.44
Nd-d-6	0.135	0.49	7.99	1.31
Nd-d-7	0.145	0.36	7.84	1.23
Nd-d-8	0.155	0.22	7.63	1.15
T= 35 °C				
Nd-a-0	0.000	13.01	9.90	
Nd-a-1	0.096	2.27	8.56	1.75
Nd-a-2	0.100	2.13	8.52	1.69
Nd-a-3	0.106	1.54	8.36	1.64
Nd-a-4	0.112	0.95	8.14	1.59
Nd-a-5	0.116	1.07	8.20	1.53
Nd-a-6	0.122	0.89	8.11	1.47
Nd-a-7	0.135	0.73	8.02	1.33
Nd-a-8	0.145	0.41	7.75	1.26
Nd-a-9	0.155	0.35	7.68	1.18
Nd-b-0	0.000	12.96	9.92	
Nd-b-1	0.094	2.48	8.60	1.75
Nd-b-2	0.100	1.08	8.20	1.73
Nd-b-3	0.107	0.78	8.05	1.65
Nd-b-4	0.134	0.47	7.81	1.34
Nd-c-0	0.000	12.99	9.90	

Nd-c-1	0.084	4.53	8.93	1.81
Nd-c-2	0.093	2.60	8.62	1.76
Nd-c-3	0.100	0.82	8.07	1.77
Nd-c-4	0.107	0.69	7.99	1.67
Nd-c-5	0.134	0.43	7.78	1.36
Nd-d-0	0.000	12.99	9.90	
Nd-d-1	0.086	3.17	8.73	1.91
Nd-d-2	0.100	0.82	8.07	1.81
Nd-d-3	0.107	0.63	7.95	1.72
Nd-d-4	0.134	0.51	7.86	1.38
T= 50 °C				
Nd-a-0	0.000	10.48	9.55	
Nd-a-1	0.070	4.91	8.91	2.21
Nd-a-2	0.073	4.44	8.85	2.19
Nd-a-3	0.077	4.01	8.79	2.15
Nd-a-4	0.080	4.21	8.82	2.05
Nd-a-5	0.084	2.85	8.60	2.14
Nd-a-6	0.088	2.23	8.47	2.12
Nd-a-7	0.094	1.28	8.20	2.12
Nd-a-8	0.097	0.93	8.05	2.10
Nd-a-9	0.100	0.54	7.81	2.07
Nd-a-10	0.107	0.33	7.58	1.97
Nd-a-11	0.133	0.23	7.43	1.59
Nd-b-0	0.000	10.00	9.49	
Nd-b-1	0.080	3.20	8.66	1.89
Nd-b-2	0.084	2.71	8.58	1.87
Nd-b-3	0.088	2.19	8.47	1.86
Nd-b-4	0.094	1.24	8.19	1.87
Nd-b-5	0.097	0.78	7.97	1.87
Nd-b-6	0.100	0.39	7.65	1.85

Nd-b-7	0.107	0.22	7.39	1.76
Nd-b-8	0.134	0.20	7.36	1.41
Nd-c-0	0.000	11.11	9.63	
Nd-c-1	0.101	5.34	8.97	1.86
Nd-c-2	0.105	5.00	8.92	1.84
Nd-c-3	0.111	4.67	8.88	1.79
Nd-c-4	0.117	3.87	8.77	1.78
Nd-c-5	0.121	3.34	8.69	1.78
Nd-c-6	0.127	2.53	8.54	1.77
Nd-c-7	0.133	1.75	8.35	1.76
Nd-c-8	0.141	0.90	8.04	1.72
Nd-c-9	0.146	0.54	7.81	1.70
Nd-c-10	0.151	0.30	7.53	1.66
Nd-c-11	0.161	0.12	7.12	1.58
Nd-c-12	0.201	0.06	6.76	1.27
Nd-d-0	0.000	10.15	9.51	
Nd-d-1	0.097	0.83	8.00	1.93
Nd-d-2	0.101	0.56	7.82	1.89
Nd-d-3	0.106	0.38	7.64	1.82
Nd-d-4	0.112	0.27	7.49	1.73
Nd-d-5	0.116	0.18	7.30	1.69
Nd-d-6	0.122	0.15	7.23	1.61
Nd-d-7	0.129	0.10	7.05	1.53
Nd-d-8	0.135	0.10	7.01	1.46
Nd-d-9	0.140	0.09	6.99	1.41
Nd-d-10	0.145	0.07	6.86	1.36
Nd-d-11	0.154	0.06	6.80	1.28
T= 75 °C				
Nd-a-0	0.000	5.37	8.99	
Nd-a-1	0.091	0.37	7.61	2.22

Nd-a-2	0.096	0.16	7.21	2.16
Nd-a-3	0.100	0.09	6.96	2.10
Nd-a-4	0.106	0.06	6.76	1.99
Nd-a-5	0.112	0.05	6.66	1.90
Nd-a-6	0.116	0.05	6.64	1.83
Nd-a-7	0.122	0.04	6.56	1.73
Nd-a-8	0.127	0.04	6.52	1.66
Nd-a-9	0.135	0.04	6.46	1.57
Nd-a-10	0.140	0.03	6.40	1.51
Nd-a-11	0.145	0.03	6.41	1.47
Nd-a-12	0.154	0.03	6.33	1.38
Nd-b-0	0.000	6.05	9.08	
Nd-b-1	0.080	1.33	8.21	2.71
Nd-b-2	0.084	1.04	8.09	2.69
Nd-b-3	0.088	0.72	7.92	2.68
Nd-b-4	0.094	0.32	7.55	2.63
Nd-b-5	0.097	0.18	7.27	2.60
Nd-b-6	0.100	0.09	6.97	2.55
Nd-b-7	0.107	0.06	6.71	2.41
Nd-c-0	0.000	6.12	9.09	
Nd-c-1	0.078	0.97	8.06	2.79
Nd-c-2	0.081	0.82	7.98	2.70
Nd-c-3	0.085	0.33	7.56	2.70
Nd-c-4	0.091	0.19	7.30	2.65
Nd-c-5	0.094	0.09	6.93	2.60
Nd-c-6	0.097	0.06	6.71	2.44
Nd-d-0	0.000	6.21	9.10	
Nd-d-1	0.084	1.16	8.14	2.75
Nd-d-2	0.088	0.93	8.04	2.72
Nd-d-3	0.094	0.59	7.83	2.67

Nd-d-4	0.097	0.37	7.61	2.65
Nd-d-5	0.100	0.33	7.56	2.58
Nd-d-6	0.107	0.22	7.37	2.46

[1]Ratio of absorbance intensity of I^{2-} and HI^{-} species at their maximum absorbance wavelengths.
[2]Measured solution pH by UV-vis.
[3]Average ligand number represents the mean number of the OH^{-} bound to Nd^{3+}.
